# Development and validation of a prognostic COVID-19 severity assessment (COSA) score and machine learning models for patient triage at a tertiary hospital

**DOI:** 10.1186/s12967-021-02720-w

**Published:** 2021-02-05

**Authors:** Verena Schöning, Evangelia Liakoni, Christine Baumgartner, Aristomenis K. Exadaktylos, Wolf E. Hautz, Andrew Atkinson, Felix Hammann

**Affiliations:** 1 Clinical Pharmacology and Toxicology, Department of General Internal Medicine, Inselspital, Bern University Hospital, University of Bern, Bern, Switzerland; 2 Department of General Internal Medicine, Inselspital, Bern University Hospital, University of Bern, Bern, Switzerland; 3 Department of Emergency Medicine, Inselspital, Bern University Hospital, University of Bern, Bern, Switzerland; 4 Pediatric Pharmacology and Pharmacometrics Research Group, University Children’s Hospital Basel, Basel, Switzerland; 5 Department of Infectious Diseases, Inselspital, Bern University Hospital, University of Bern, Bern, Switzerland

**Keywords:** SARS-CoV-2, Critical illness, Risk stratification, Statistical learning, Artificial intelligence

## Abstract

**Background:**

Clinical risk scores and machine learning models based on routine laboratory values could assist in automated early identification of severe acute respiratory syndrome coronavirus 2 (SARS-CoV-2) patients at risk for severe clinical outcomes. They can guide patient triage, inform allocation of health care resources, and contribute to the improvement of clinical outcomes.

**Methods:**

In- and out-patients tested positive for SARS-CoV-2 at the Insel Hospital Group Bern, Switzerland, between February 1st and August 31st (‘first wave’, n = 198) and September 1st through November 16th 2020 (‘second wave’, n = 459) were used as training and prospective validation cohort, respectively. A clinical risk stratification score and machine learning (ML) models were developed using demographic data, medical history, and laboratory values taken up to 3 days before, or 1 day after, positive testing to predict severe outcomes of hospitalization (a composite endpoint of admission to intensive care, or death from any cause). Test accuracy was assessed using the area under the receiver operating characteristic curve (AUROC).

**Results:**

Sex, C-reactive protein, sodium, hemoglobin, glomerular filtration rate, glucose, and leucocytes around the time of first positive testing (− 3 to + 1 days) were the most predictive parameters. AUROC of the risk stratification score on training data (AUROC = 0.94, positive predictive value (PPV) = 0.97, negative predictive value (NPV) = 0.80) were comparable to the prospective validation cohort (AUROC = 0.85, PPV = 0.91, NPV = 0.81). The most successful ML algorithm with respect to AUROC was support vector machines (median = 0.96, interquartile range = 0.85–0.99, PPV = 0.90, NPV = 0.58).

**Conclusion:**

With a small set of easily obtainable parameters, both the clinical risk stratification score and the ML models were predictive for severe outcomes at our tertiary hospital center, and performed well in prospective validation.

## Background

Coronavirus disease 19 (COVID-19) is an infectious disease caused by the severe acute respiratory syndrome coronavirus 2 (SARS-CoV-2). First identified in Wuhan, China, in December 2019, [1] it spread globally and resulted in a pandemic with over 55 million cases and over 1.6 million deaths by early of November 2020 [2].

A large proportion, approximately 40–45%, of infected patients show little or no symptoms, [3] and, depending on the study, ICU admission rates are estimated between 5 and about 30% of hospitalized patients [4]. Development of individual clinical courses is not always predictable and, together with the sheer number of patients at-risk for critical or fatal outcomes, this poses challenges in patient triage, allocation of health care resources, and utilization of intensive care facilities [5, 6].

There have been efforts to develop predictive scores and algorithms to address these needs. Given the heterogeneity in symptoms on presentation and the potential outcomes, [7, 8] it is not surprising that many of the tools proposed so far have relied on complex sets of parameters or specialized laboratory markers. The recently published COVID-19 Acuity Score (CoVA) developed from electronic health record (EHR) data of out-patients in the Boston area (n = 9381), for instance, contains 30 items, including presence of intracranial hemorrhage and hematological malignancy [9]. While it has been shown to predict hospitalization, critical illness, and death with accuracies of 0.76–0.93 for the area under the receiver operating characteristic (AUROC, ranging from 0 to 1 with a value of 0.5 indicating no class separation above randomness), only 15% (n = 1404) of the cases in the development cohort had a confirmed positive SARS-CoV-2 test. Del Valle et al*.* described correlation between prognosis and serum interleukin (IL)-6, IL-8, tumor necrosis factor (TNF)-α and IL-1β, which, though predictive, are expensive non-routine tests [10]. Several tools also rely on chest x-ray findings, some of which use machine learning (ML) to automatically classify digital images [9, 11, 12]. The value of chest x-rays, however, has been called into question, as no lesions specific for COVID-19 have so far been identified, and images may appear normal despite pulmonary symptoms [13].

The aim of the present study was to develop easily deployable screening tools for early identification of COVID-19 patients at risk for severe outcomes, defined as a composite endpoint consisting of either requiring treatment in an intensive care unit (ICU), or death from any cause. The tools include a clinical prediction rule scoring system intended for bedside use by practitioners, and several ML models suitable for deployment in EHR systems for automated monitoring. Potential applications include real-time screening of in-patients to gauge future demand for intensive care, and decision support at point-of-care in patient triage. The investigated covariates, e.g. medical history, patient demographics, and laboratory values often routinely assessed on admission, such as blood glucose, sodium, or C-reactive protein, were chosen due to their ease of availability. We trained models using in- and out-patients seen at our tertiary hospital during spring and summer of 2020 (‘first wave’). To confirm the findings were generalizable, we validated them prospectively using the cases of the ‘second wave’ during autumn 2020.

## Methods

### Study population and training cohort

**Fig. 1:**
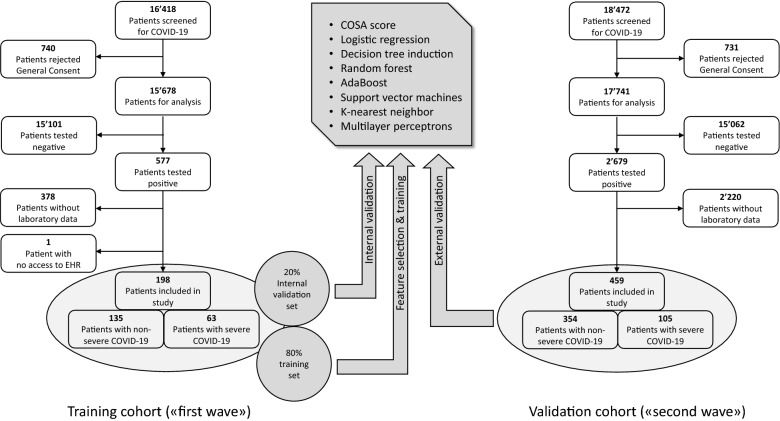
Flowchart of training and validation cohort used for model and score development

The study was approved by the Cantonal Ethics Committee of Bern (Project-ID 2020-00973), and carried out at the Insel Hospital Group (IHG), a tertiary hospital and the biggest health care provider in Switzerland with six locations and about 860000 patients treated per year. For the training cohort, we considered all individuals who tested positive for SARS-CoV-2 at the IHG between February 1st through August 31st 2020–covering the ‘first wave’ of COVID-19 in the country, and who did not reject the general research consent. For patients with no registered general research consent status, a waiver of consent was granted by the ethics committee. Patients who objected to the general research consent of the IHG, or who tested negative for SARS-CoV-2, were excluded from the study. Participation in other trials (incl. COVID-19 related treatment studies) was not an exclusion criterion and was not recorded separately. For SARS-CoV-2 detection, a reverse-transcriptase polymerase chain reaction (RT-PCR) assay was used on nasopharyngeal swabs as a diagnostic test. Detailed information on the selection of the study population is provided in Fig. 1. All patients were discharged or had died by the time of model development.

For the development of the score and the ML models, patients were classified according to their disease severity (the primary outcome), with the worst outcome at any point after the first positive diagnosis determining the class:

- *Non*-*severe* Patients who tested positive for SARS-CoV-2, but were neither admitted to the ICU nor died of any cause during their hospital stay (classified as ‘negative’).
- *Severe* Composite outcome for patients who tested positive for SARS-CoV-2 and required ICU admission at any stage during the disease and/or died of any cause during their hospital stay (classified as ‘positive’).

Given that the study was retrospective and observational, sample sizes were dictated by the dynamics of the pandemic in the greater Bern region. Consequently, no formal power calculations were performed a priori.

### Validation cohort

As independent time-sliced validation cohort, patients from the ‘second wave’ (first positive test for SARS-CoV-2 between September 1st and November 16th 2020) were identified. Exclusion criteria and allocation to severity group were the same as for the training cohort. For patients from the validation cohort with no registered consent status, a waiver of consent was granted by the ethics committee. All patients were discharged or had died by the time the validation was performed.

### Data preparation

We selected the 20 most frequently measured laboratory values (see Additional file 1: Figure S1–1). For highly correlated variables, the most easily obtainable one was used in the study (e.g. for erythrocyte count and hemoglobin, the latter was chosen because its determination does not require flowcytometric methods). We then selected those variables that were either positively or negatively correlated with the outcome (severe or non-severe COVID-19) as identified by pairwise Pearson's correlation.

Since our goal was to develop tools for early identification of at-risk patients, we only considered laboratory values from the 3 days leading up to the first positive RT-PCR, as well those from the day following the positive test–corresponding to the intended time of use of the score and models. If multiple measurements were available for a given parameter, we chose the most extreme values so as to minimize bias from treatment effects. Missing data (3% of all data points, most frequently estimated glomerular filtration rate (eGFR)) were imputed using the k-nearest neighbor algorithm.

Demographic data were extracted from the EHR and included age at time of COVID-19 test, sex, weight, height, and body mass index (BMI). Again, for time-varying covariates, we chose the values available closest to the first positive RT-PCR test. Two authors manually screened the EHR for the medical history of patients in the training cohort. Specifically, we assessed substance use (nicotine, alcohol), cardiovascular diseases (arterial hypertension, coronary and chronic heart disease, stroke, other cardiovascular disease), pulmonary diseases (asthma, chronic obstructive pulmonary disease (COPD), other pulmonary disease), type II diabetes, and cancer.

As ethnicity is unfortunately not systematically collected in the EHR, and we were not able to retrospectively obtain these data, we were unable to evaluate its effect on disease severity. Furthermore, even though other studies suggested the importance of proteomics and metabolomics [14–16], these data were also not available for the patients anaylzed, and could therefore not be included in the research.

### COVID-19 severity assessment score

The training cohort was randomly divided into an internal training (80%) and an internal hold-out validation (20%) set, stratified for severe and non-severe cases in each set. Using the training set, the parameters were plotted against the severity using the local polynomial regression fitting (LOESS) function [17]. In combination with the splits provided by the Decision Trees (DTI; see below), these graphs were used to define the cut-offs for continuous parameters. For the included laboratory values, cut-offs were set as close as possible to the upper or lower normal range values, to allow for an early identification of patients at risk. Based on the cut-offs, the continuous parameters (i.e. laboratory values) were converted into categorical parameters. For categorical parameters (e.g. sex), the existing levels were kept. Score points of each level and each parameter were obtained by fitting a logistic regression model. We examined several different parameters and combinations thereof based on mechanistical plausibility. As we aimed to develop an easy and quick score with as few parameters as possible, we excluded parameters, which were only weakly correlated with the investigated outcome (Pearson correlation coefficients between -0.4 and 0.4, see Additional file 1: Figure S1–2), as we expected them to not improve the overall performance of the score as assessed by the AUROC. From the remaining parameters, we excluded parameters, which were correlated (hemoglobin was correlated to erythrocytes, hematocrit, and mean corpuscular haemoglobin concentration) or described the same organ system (eGFR and creatinine). For two parameters (red cell distribution width and mean platelet volume) no suitable cut-offs could be defined and so they were excluded. The total score was calculated for each patient in the training and internal validation set.

In a second step, the probability of a severe outcome was determined by fitting the total multivariable score to the observed outcome using logistic regression. To quantify the predictive value of the score, the AUROC was calculated. As the results of the fitting depend on the splitting of the training and validation set, the steps described above were repeated with 30-fold cross-validation (we chose this number to obtain the same overall number of repeats as with the repeated cross-validation of the ML models, see below). The final score points with which each parameter contributes to the total score was the median of the 30 folds. This step also served as internal cross-validation. As external validation, we evaluated patients from the validation cohort (patients tested positive for COVID-19 between September 1st and November 16th, 2020). We used the above score, predicted the outcome for the external validation cohort, and compared this with the actual outcome, then calculated the AUROC.

### Machine learning models

In a further step, different statistical and ML models were fitted to the dataset. First, we used standard multivariate logistic regression (LogReg).Then we fitted machine learning models, which mimic human decision processes, either rule-based as decision tree induction (DTI) using a variation of classification and regression trees (CART) and random forest (RF, an aggregation of multiple decision trees), or based on similarities such as k-nearest neighbor (kNN). Lastly, we applied high-dimensional, complex algorithms known to generate robust classification models such as AdaBoost, support vector machines (SVM) with linear kernel, and multilayer perceptrons (MLP) [18]. Parameter values were scaled to range between 0 to 1, except for DTI and RF, where the original parameter values were used. Model weights were calculated to account for the slightly unbalanced outcomes. All models were trained using internal three times repeated tenfold cross-validation. An external prospective validation of these models was performed on the validation cohort to compare performance parameters of the internal and the external validations.

### Software and statistical tests

Data wrangling, analysis and visualization was performed in GNU R (version 4.0.2, R Foundation for Statistical Computing, http://www.R-project.org, Vienna, Austria). Standard statistics, e.g. logistic regression, LOESS function, Chi square test, Wilcoxon rank sum test, were conducted using the *stats* package (version 4.0.2). For ML models the packages *caret* (version 6.0–86), *rpart* (version 4.1–15), *randomForest* (version 4.6–14), *DMwR* (version 0.4.1), *e1071* (version 1.7–4), and *RSNNS* (version 0.4–12) were used.

Statistical significance levels were determined using the Wilcoxon rank sum test for non-normally distributed parameters, as confirmed by the Shapiro-Wilk test, and the Chi square test for categorical parameters.

## Results

### Study population and training cohort

A total of 16418 patients were screened for SARS-CoV-2 between February 1st and August 31st 2020, of which 740 rejected general research consent. Of the 577 eligible patients testing positive (419 out-patients and 158 requiring hospitalization), sufficient data was available for 198 (no laboratory analysis performed in 378 cases and no access to the EHR in one case) which made up the final training cohort, and were grouped based on the outcome in 63 severe and 135 non-severe cases (Fig. 1). Mean time to ICU admission in the severe group was 1.9 days (range: 3 days before COVID-19 test and 45 days after positive RT-PCR test), and the mean time to death (n = 25) was 22.1 days (range: 1 to 79 days after positive RT-PCR test).

As shown in Table 1, patients with severe disease were predominantly older and male. While there was no difference in substance use (nicotine or alcohol) or pulmonary disorders, there was an association with more cardiovascular comorbidities (arterial hypertension, cardiomyopathy and congestive heart failure, and coronary heart disease, but not stroke or other cardiovascular diseases) and type II diabetes. We saw no difference in the prevalence of cancer.

**Table 1:** General characteristics and laboratory parameters of the training cohort (n = 198) and validation cohort (n = 459)

	Training cohort (N = 198)			Validation cohort (N = 459)		
	Severe (N = 63)	Non-severe (N = 135)	P value	Severe (N = 105)	Non-severe (N = 354)	P value
Demographics						
Age (years)						
Median (IQR)	65.0 (53.5, 79.5)	54.0 (36.0, 71.5)	< *0.002*^*a*^	69.0 (61.0, 79.0)	62.0 (46.0, 75.0)	< *0.002*^a^
Sex						
Female, n (%)	11 (17.46%)	69 (51.11%)	< *0.002*^*b*^	33 (31.43%)	150 (42.49%)	0.055^b^
Hospitalization						
Inpatients, n (%)	61 (96.83%)	84 (62.22%)	< *0.002*^*b*^	102 (97.14%)	216 (61.19%)	< *0.002*^*b*^
Deaths						
Deceased, n (%)	25 (39.68%)	0 (0.00%)	< *0.002*^*b*^	33 (31.43%)	0 (0.00%)	< *0.002*^*b*^
Weight (kg)						
Median (IQR)	83.00 (70.40, 97.00)	75.65 (62.00, 87.00)	0.241^a^	80.60 (70.00, 90.15)	77.20 (65.70, 87.00)	0.989^a^
Height (cm)						
Median (IQR)	172.00 (165.00, 178.25)	170.00 (163.00, 177.00)	*0.012*^*a*^	170.00 (165.00, 176.00)	170.00 (164.00, 176.00)	*0.037*^*a*^
Body Mass Index (BMI, kg/m2)						
Median (IQR)	27.62 (24.80, 31.57)	25.82 (22.52, 28.95)	*0.018*^*a*^	28.10 (24.95, 32.62)	26.23 (23.62, 29.59)	< *0.002*^*a*^
Laboratory parameters						
Maximal CRP levels						
Median (IQR)	176.00 (92.75, 279.75)	22.00 (6.00, 67.00)	< *0.002*^*a*^	146.00 (72.75, 228.25)	35.00 (8.00, 89.00)	< *0.002*^*a*^
Maximal sodium levels						
Median (IQR)	142.00 (139.50, 146.00)	139.00 (136.00, 141.00)	< *0.002*^*a*^	142.00 (139.00, 145.00)	139.00 (137.00, 141.00)	< *0.002*^*a*^
Minimal hemoglobin levels						
Median (IQR)	96.00 (78.25, 115.00)	132.00 (119.00, 141.00)	< *0.002*^*a*^	110.00 (86.00, 122.00)	128.00 (116.00, 141.00)	< *0.002*^*a*^
Minimal glomerular filtration rate (GFR) values						
Median (IQR)	53.00 (25.00, 85.50)	87.00 (72.00, 104.00)	< *0.002*^*a*^	62.50 (32.50, 81.00)	82.00 (59.00, 98.00)	< *0.002*^*a*^
Maximal glucose values						
Median (IQR)	10.10 (8.00, 12.75)	6.10 (5.48, 7.62)	< *0.002*^*a*^	10.70 (8.30, 13.50)	6.42 (5.60, 8.20)	< *0.002*^*a*^
Maximal leukocytes values						
Median (IQR)	10.60 (7.83, 17.55)	5.92 (4.70, 7.77)	< *0.002*^*a*^	12.30 (8.95, 16.00)	6.73 (4.97, 8.78)	< *0.002*^*a*^
Comorbidities						
Substance use						
Smoker, n (%)	11 (17.46%)	16 (11.85%)	0.400^b^	–	–	–
Alcohol, n (%)	5 (7.94%)	11 (8.15%)	1^b^	–	–	–
Cardiovascular disorders						
Cardiovascular disorders, overall, n (%)	47 (74.60%)	56 (41.48%)	< *0.002*^*b*^	–	–	–
Arterial hypertension, n (%)	35 (55.56%)	43 (31.85%)	*0.003*^*b*^	–	–	–
Coronary heart disease, n (%)	13 (20.63%)	6 (4.44%)	< *0.002*^*b*^	–	–	–
Congestive heart failure and cardiomyopathy, n (%)	16 (25.40%)	14 (10.37%)	*0.011*^*b*^	–	–	–
Stroke, n (%)	8 (12.70%)	11 (8.15%)	0.451^b^	–	–	–
Other, n (%)	16 (25.40%)	24 (17.78%)	0.292^b^	–	–	–
Pulmonary disorders						
Pulmonary disorders, overall, n (%)	18 (28.57%)	35 (25.93%)	0.826^b^	–	–	–
Asthma, n (%)	4 (6.35%)	15 (11.11%)	0.423^b^	–	–	–
Chronic obstructive pulmonary disease (COPD), n (%)	6 (9.52%)	5 (3.70%)	0.183^b^	–	–	–
Other, n (%)	10 (15.87%)	19 (14.07%)	0.906^b^	–	–	–
Other disorders						
Type II diabetes (incl. prediabetes), n (%)	22 (34.92%)	20 (14.81%)	*0.002*^*b*^	–	–	–
Cancer, n (%)	6 (9.52%)	16 (11.85%)	0.808^b^	–	–	–

Laboratory values were considered from 3 day prior to until 1 day after the first positive SARS-CoV-2 PCR test result; italic numbers indicate significant differences (p < 0.05) between severe and non-severe cases.

*IQR* interquartile range

^a^Wilcoxon rank sum test

^b^Chi Square test

After parameter selection, the following laboratory parameters were ultimately included as laboratory covariates: C-reactive protein (CRP), sodium, hemoglobin, estimated glomerular filtration rate (eGFR) according to the Chronic Kidney Disease Epidemiology Collaboration (CKD-EPI) equation (provided by the EHR system), glucose, and leucocytes. For demographics, the sex of the patient was added as a categorical variable. Even though age and obesity (as BMI) are generally considered as risk factors for a severe COVID-19, the correlations were not informative in our models. Strikingly, no item in the patients’ medical history carried enough information to be included in the final predictors.

### Validation cohort

During the period between September 1st and November 16th 2020, 18′472 patients received SARS-CoV-2 testing. General research consent was rejected by 731. Of the 2′679 eligible patients testing positive, sufficient data was available for 459 patients (141 out-patients and 318 in-patients). The final validation data set consisted of 105 severe and 354 non-severe cases. Detailed information is presented in Table 1.

### COVID-19 severity assessment score

**Fig. 2:**
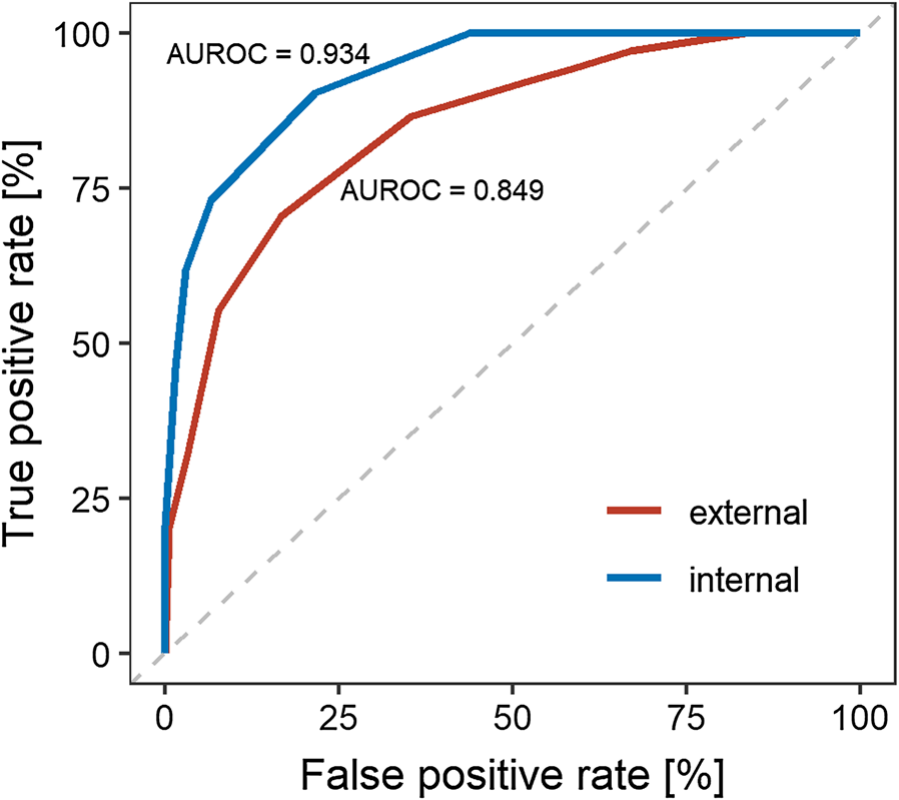
Area under the receiver operating characteristic (AUROC) of the COVID-19 severity assessment (COSA) score. Training cohort (red, internal) and validation cohort (blue, external)

**Fig. 3:**
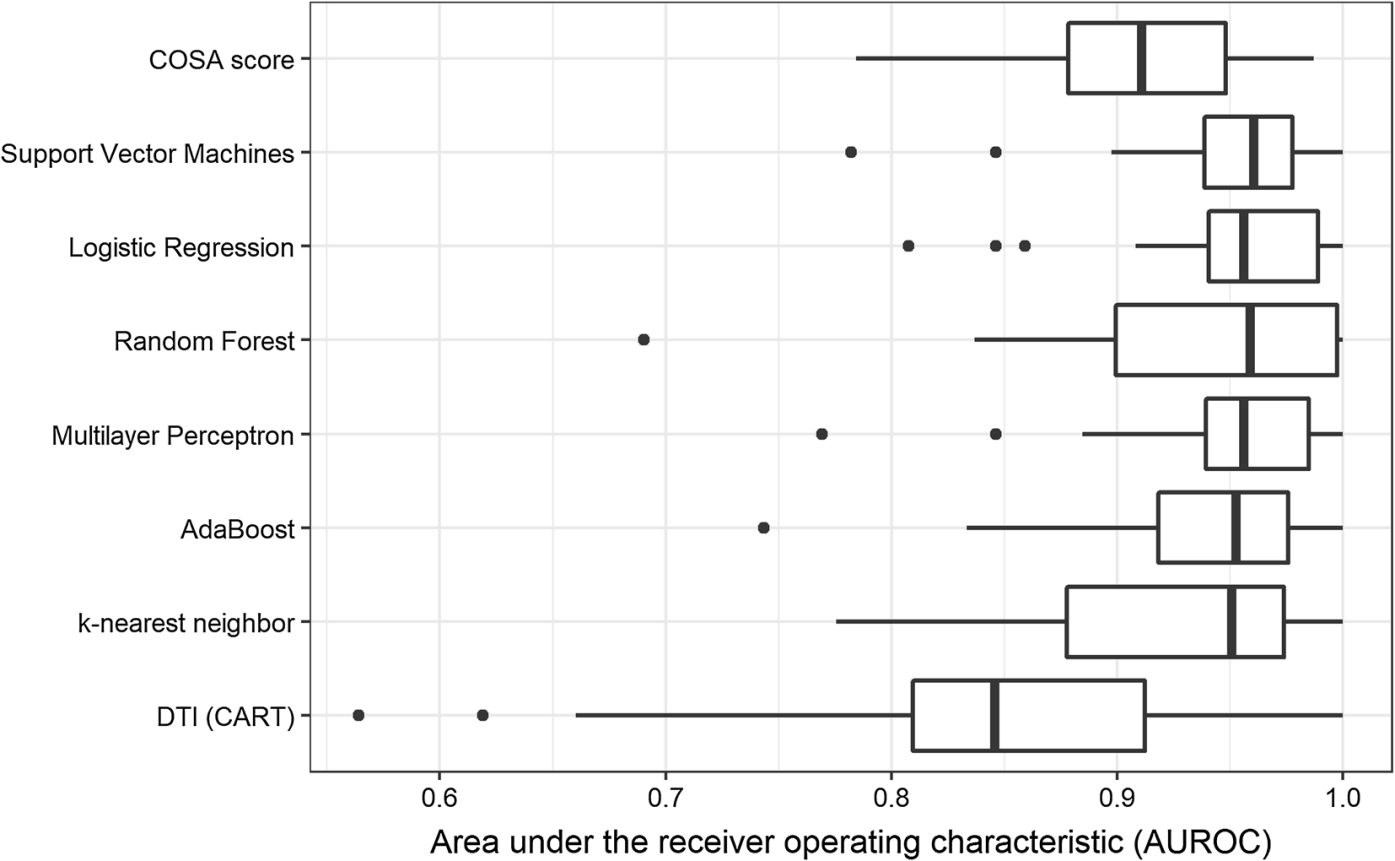
Performance of the clinical risk stratification COVID-19 severity assessment (COSA) score and machine learning models. Performance on training data as measured by area under the receiver operating characteristic. DTI (CART): decision tree induction with classification and regression trees. The lower and upper hinges of the box correspond to interquartile ranges (IQR). The upper and lower whisker extends from the hinge to the largest and smallest value, respectively, no further than 1.5 * IQR from the hinge. Data beyond the end of the whiskers are plotted individually

Of the commonly measured laboratory parameters included in the predictive models, sodium, CRP, glucose and leucocytes were positively correlated with severe COVID-19, indicating that higher values pose a higher risk for worse outcomes. Hemoglobin and eGFR were negatively correlated with severe outcomes. The AUROC for the 30 internal validation folds ranged from 0.84 to 0.98 (Fig. 3), indicating that the chosen parameters and cut-offs were good predictors for the endpoint. The final score allocation is shown in Table 1 with the comparison of the AUROC for the complete study and validation cohort in Fig. 2. The percentage of severe and non-severe courses per total score of all patients with laboratory values (including those with missing values) for the study and validation cohort is shown in Additional file 2: Figure S2. Based on the distribution, a total score of up to 3 was associated with no cases of severe COVID-19 in the training cohort, (i.e. a specificity of 100%) thus representing a low risk. A score of 4 or 5 was associated with < 50% severe cases thus indicating a moderate risk, while 6 to 7 points were associated with > 50% severe cases (high risk), and 8 or more points correlated with a very high risk to develop a severe COVID-19 (100% of the patients with severe disease, i.e. 100% sensitivity) (Table 2).

**Table 2:** COVID-19 severity assessment (COSA) score parameters and evaluation

Parameter	Value	Score points
Sex	Male	1
CRP	≥ 25 mg/L	3
Sodium	≥ 144 mmol/L	2
Hemoglobin	≤ 100 g/L	1
eGFR according to CKD-EPI	≤ 75 mL/min	1
Glucose	≥ 8.6 mmol/L	1
Leucocytes	≥ 10 G/L	1

Score evaluation			
Total score per patient	Category	% patients (training, validation)	% severe outcome (training, validation)
0–3 points:	Low risk for severe COVID-19 (< 5%)	37.9, 38.4%	0, 4.5%
4–5 points:	Moderate risk for severe COVID-19 (< 50%)	34.8, 32.5%	24.6, 15.4%
6–7 points:	High risk for severe COVID-19 (> 50%)	12.1, 19.4%	70.8, 46.1%
8–10 points:	Very high risk for severe COVID-19 (> 90%)	15.2, 9.6%	96.7, 75.0%

Laboratory parameters are maximal (C-reactive protein (CRP), sodium, glucose, leucocytes) or minimal (estimated glomerular filtration rate (eGFR) according to the Chronic Kidney Disease Epidemiology Collaboration (CKD-EPI)) values 3 days prior to and up to 1 day after first positive SARS-CoV-2 testing. % patients: fraction of patients per category, % severe: fraction of patients with severe outcomes per category

External prospective validation was done without changes to the original models, i.e. with no recalibration. The score performed consistent with findings from the internal validation: the measured metrics in the validation cohort were lower (AUROC 0.85, positive prediction value (PPV) 0.91, negative prediction value (NPV) 0.81) than in the training cohort (AUROC 0.94, PPV 0.97, NPV 0.80), but still showed a very good predictivity. No patient with 0 score points developed a severe COVID-19, patients with 6 or less score points developed a severe course in less than 50% of the cases, while at 7 and 8 score points more than 50% showed a severe COVID-19. Patients with 9 or more score points were severely ill in 90% of the cases. The correlation coefficient of the chosen laboratory parameters was in general lower in the validation cohort than in the training cohort, but the ranking of the parameters was in good agreement with the training cohort (see Additional file 1: Figure S1–3).

### Machine learning models

The results of the internal validation of the different ML models are shown in Fig. 3, with median AUROC values ranging from 0.86 (DTI) to 0.96 (SVM). The employed ML models were well suited to distinguish between severe and non-severe COVID-19 with the laboratory parameters provided. The results of the external validation of the original models in Additional file 3: Figure S3 suggest that the ML algorithms produced predictive and generalizable models.

## Discussion

In this study, we created a clinical score and ML models that accurately predicted the likelihood of severe disease courses (defined as requiring ICU admission at any stage during the disease and/or death of any cause during the study period) for SARS-CoV-2 positive patients at the largest hospital group in Switzerland during the country’s ‘first wave’ of the COVID-19 pandemic. An external validation using the larger ‘second wave’ patient cohort also confirmed the prognostic value of the score and models, and thus the generalizability.

The most predictive risk factors were male sex, low hemoglobin (< 100 g/L), elevation of inflammatory parameters (CRP > 25 mg/L, leucocyte counts > 10 G/L), hyperglycemia (> 10 mmol/L), and impaired renal function (eGFR < 75 mL/min, sodium > 144 mmol/L). Since most of those parameters are readily available/commonly measured at presentation, the score can provide early-stage guidance regarding patient triage, thus contributing to the improvement of outcomes by enabling timely and targeted use of health care resources for patients at risk for severe clinical courses.

Regarding the individual laboratory score components, an increase in inflammatory parameters as a predictor of severe disease is mechanistically plausible and well documented [19–22]. Similar to other infections, loss of glycemic control has been reported in COVID-19 patients (with elevated blood glucose increasing the risk of SARS-CoV-2 infection), and also that well controlled diabetes mellitus correlates with favorable outcomes [23, 24]. A systematic review and meta-analysis further corroborated these findings [25]. There is ample evidence that end-stage kidney disease and renal impairment (as reflected by eGFR in our analysis) are prognostic of more severe disease, with case fatality rates on ICU of up to 50% [26, 27]. Similarly, electrolyte disorders like hypernatremia have been linked to increased COVID-19 related mortality, possibly in relation to increased respiratory rate or dehydration from increased body temperature [28, 29]. Finally, a systematic review and meta-analysis recently discussed the role of anemia and changes in iron metabolism, reflected by the low-normal cut-off for hemoglobin in our analysis, in the pathophysiology and disease course of COVID-19 [30].

One of the hallmarks of COVID-19 is its disproportionally high mortality in the elderly, possibly due to multimorbidity [31, 32]. More detailed surveys report an increased likelihood of death following development of symptoms in the age groups < 30 years and > 65y years [33]. It has been speculated that younger patients with severe courses experience hyperinflammatory syndromes (often referred to as ‘cytokine storms’), an IL-6 driven over-reaction of the immune system to pathogens resulting in multi-organ failure that is associated with a high mortality [34]. Several studies identified high age and obesity, which are often connected to a reduced state of health, as risk factor for severe COVID-19 [35, 36]. Therefore, these parameters are not independent risk factors in our cohort despite statistically significant differences between the means, arguably because of their correlation with multimorbidity. To avoid overfitting due to intercorrelated parameters, we rejected age and obesity (as BMI) in favor of other correlated features that also explain additional cases.

There is also a large body of evidence concerning underlying diseases as risk factors associated with critical disease and overall COVID-19 mortality [37–39]. We screened the EHRs for presence of cardiovascular comorbidities (incl. arterial hypertension and stroke events), obesity, chronic pulmonary conditions, kidney disease, cancer, diabetes mellitus, and smoking status. Of those, only cardiovascular disease (overall, hypertension, coronary heart disease, and cardiomyopathy or chronic heart failure), and type II diabetes had a prognostic value. However, none of those parameters were more predictive than male sex and laboratory values taken around the time of first RT-PCR. Hyperglycemia, anemia, and impaired renal function are signs or risk factors for deterioration and poor prognosis of these comorbidities in their own right, and may indicate that poorly controlled underlying diseases are more detrimental than the diseases themselves.

In the light of the worldwide spread of SARS-CoV-2 leading to high rate of fatalities and shortage of hospital beds in many countries, several attempts have been undertaken to create predictive scores and models for the early identification of patients at high risk. In a regularly updated systematic review by Wynants et al*.* [40], 50 prognostic models were identified, including 23 for mortality and 8 for progression to severe disease. Frequently reported prognostic variables were sex, comorbidities, CRP and creatinine. All models reported moderate to excellent predictive performance, but were judged as being at high risk of bias (e.g. due to exclusion of participants still in follow-up who didn’t develop the outcome at the end of the study, and use of the last available measurement instead of one at the time of intended use of the model), and none of them is currently recommended for use in clinical practice [40]. Recommendations for future investigations in this field include adequate inclusion/censoring and description of the study population, specification of the time horizon of the prediction, and structured reporting based on the Transparent Reporting of a multivariable prediction model for Individual Prognosis or Diagnosis (TRIPOD) guidelines [41] to enable independent validation, which we aimed to follow closely with this study.

Limitations of the current study include the small sample size, and the exclusion of patients who rejected the IHG general research consent and those with no laboratory data available. The excluded 379 individuals (65.7% of the total of 577 patients who did not reject the general research consent and were tested positive) correspond mostly to patients seen in the ambulatory COVID-19 testing facility, where the general public have access to on-demand testing. Furthermore, more of the included patients were hospitalized, while comorbidities and risk factors might differ among individuals who could be treated as out-patients. There was no specific time-to-event analysis, particularly since the data generated in the first months of the pandemic was very heterogeneous, and included external direct transfers to ICU. The score and models therefore only speak to the probability of incurring a severe outcome, not when this outcome will occur. Another limitation concerns the censoring of outcomes, given there was no explicit follow-up. While it is conceivable that out-patients in particular could have deteriorated after discharge and presented elsewhere, the large catchment area of the IHG should mitigate this effect. Additionally, the case-fatality ratios of 12.6%, and 7.2% in the training group (‘first wave’) and the prospective validation group (‘second wave’), respectively, are high compared to the Swiss national average (1.1%, https://covid19.bag.admin.ch). This hints at good coverage of outcomes in these retrospective analyses along with presence of an admission bias. We therefore suggest using the tools proposed here for projection of outcomes as discussed.

## Conclusion

In conclusion, based on the findings of the study including external validation, the COSA score and ML models based on commonly available laboratory values can help predict the likelihood of a severe clinical course early on during COVID-19 disease, thus allowing stratification to treatment regimens and identification of patients who should be put under close monitoring to detect early deterioration. Future validations could include other hospital centers as well as general practitioners.

## Supplementary Information

**Additional file 1**. Common laboratory parameters and their correlation to the outcome (severe or non-severe COVID-19).

**Additional file 2**. Score points in study and validation cohort.

**Additional file 3**. External validation metrics.

## Data Availability

An online version of the score is available at https://cptbern.github.io/cosa/. The source code and corresponding input files are available on GitHub: https://github.com/cptbern/COSAscore. The datasets used and/or analysed during the current study are available from the corresponding author on reasonable request.

## Abbreviations

AUROC: Area under the receiver operating characteristic curve
BMI: Body mass index
CART: Classification and regression trees
COPD: Chronic obstructive pulmonary disease
COSA: COVID-19 severity assessment
COVID-19: Coronavirus disease 19
CRP: C-reactive protein
DTI: Decision trees
eGFR: Estimated glomerular filtration rate
EHR: Electronic health records
ICU: Intensive care unit
IHG: Insel Hospital Group
IL: Interleukin
kNN: K-Nearest neighbor
LogReg: Logistic regression
ML: Machine learning
MLP: Mulit-layer perceptrons
NPV: Negative predictive value
PPV: Positive predictive value
RF: Random forest
RT-PCR: Reverse-transcriptase polymerase chain reaction
SARS-CoV-2: Severe acute respiratory syndrome coronavirus 2
SVM: Support vector machines
