# Supplementary material for: Development and validation of a prognostic COVID-19 severity assessment (COSA) score and machine learning models for patient triage at a tertiary hospital
Source: J Transl Med. 2021 Feb 5;19:56. doi: 10.1186/s12967-021-02720-w (PMC7862984; doi:10.1186/s12967-021-02720-w)
Supplement: Supplementary file 1 — Additional file 1. Common laboratory parameters and their correlation to the outcome (severe or non-severe COVID-19). [file 12967_2021_2720_MOESM1_ESM.pdf]

# Additional file 1

### Common laboratory parameters and their correlation to the outcome (severe or non-severe COVID-19)

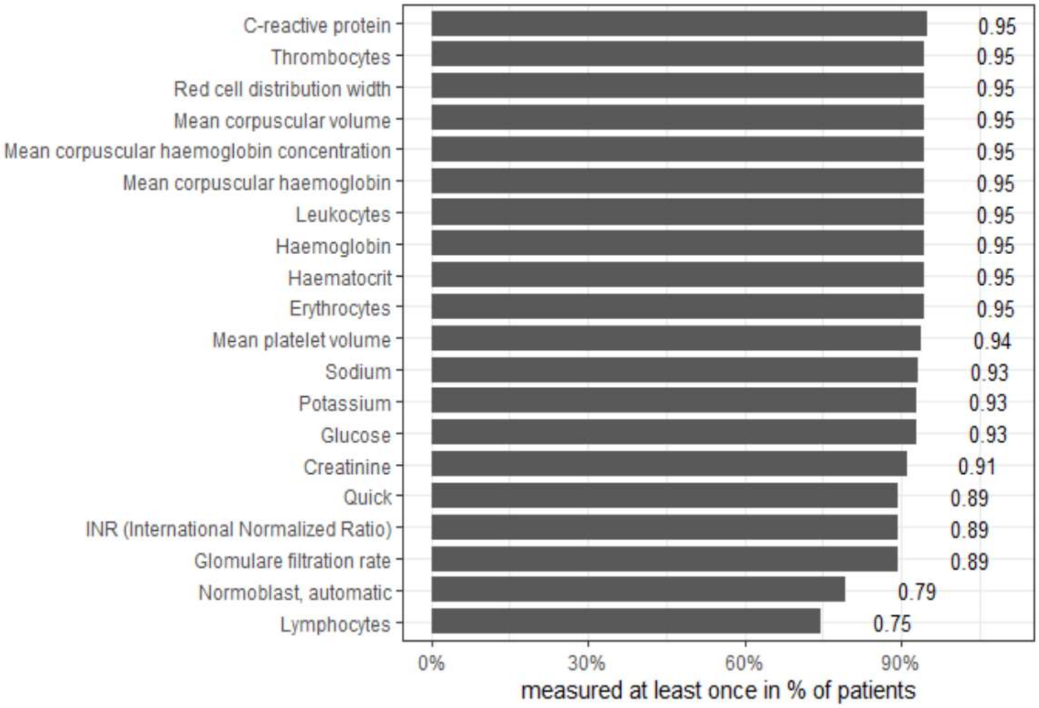


**Figure S1-1:** Overview of the most commonly tested blood parameters in patients with coronavirus disease 19 (COVID-19) at the Insel Hospital Group (IHG)

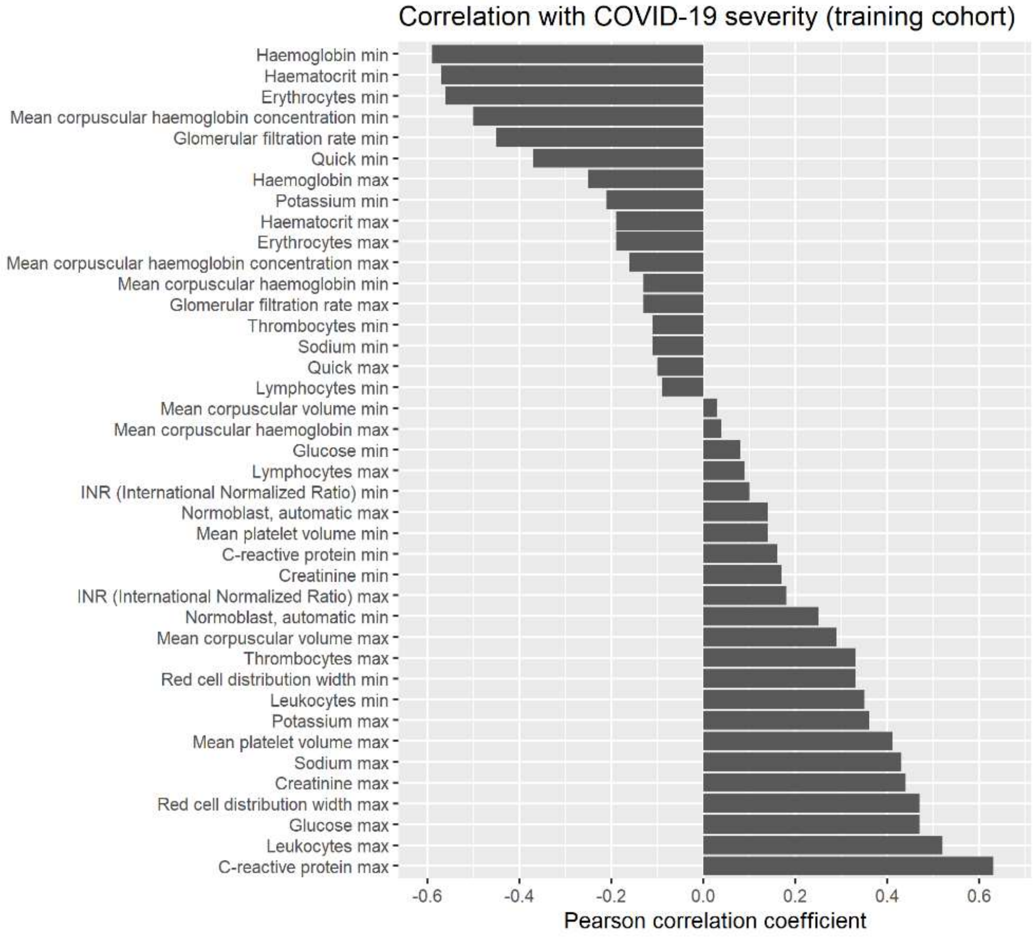


**Figure S1-2:** Correlation of the most commonly tested blood parameters with the severity of COVID-19 in the training cohort ('1st wave')

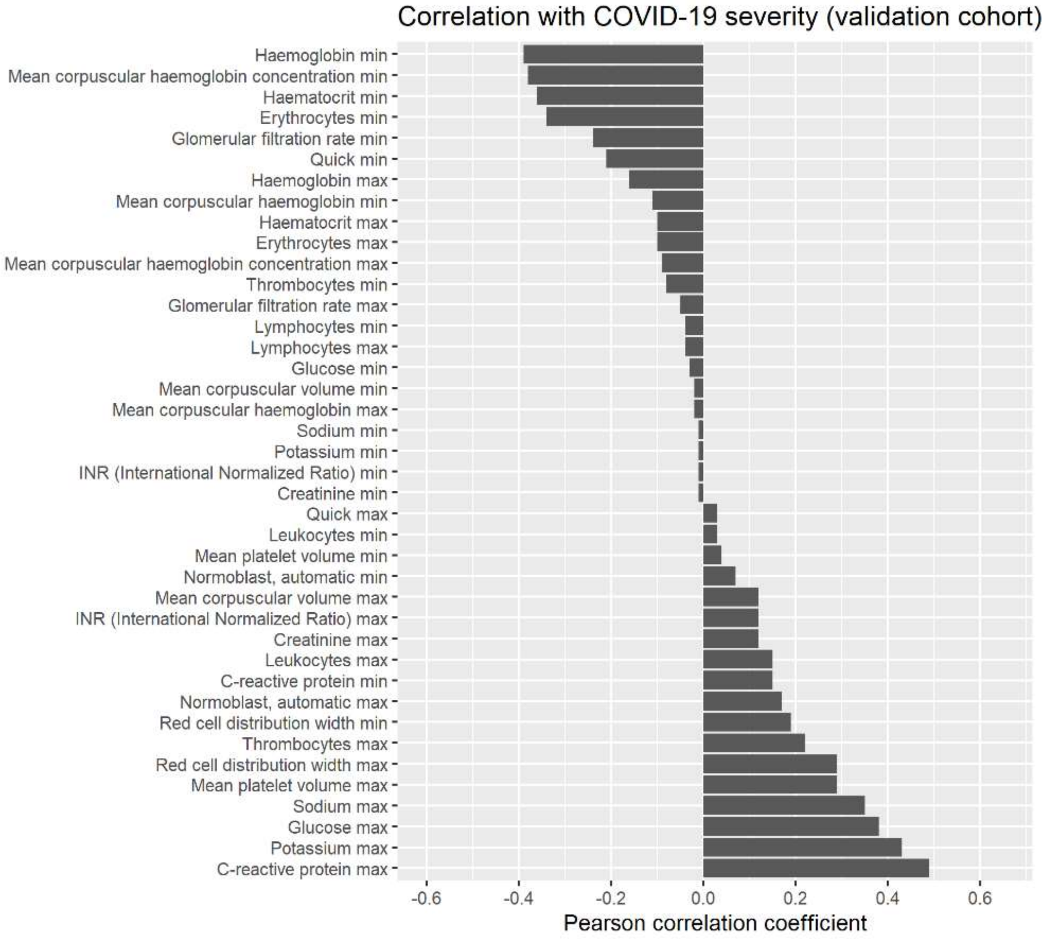


**Figure S1-3:** Correlation of the most commonly tested blood parameters with the severity of COVID-19 in the validation cohort (‘2nd wave’)
