# Supplementary material for: Development and validation of a prognostic COVID-19 severity assessment (COSA) score and machine learning models for patient triage at a tertiary hospital
Source: J Transl Med. 2021 Feb 5;19:56. doi: 10.1186/s12967-021-02720-w (PMC7862984; doi:10.1186/s12967-021-02720-w)
Supplement: Supplementary file 2 — Additional file 2. Score points in study and validation cohort. [file 12967_2021_2720_MOESM2_ESM.pdf]

Additional file 2

Score points in study and validation cohort

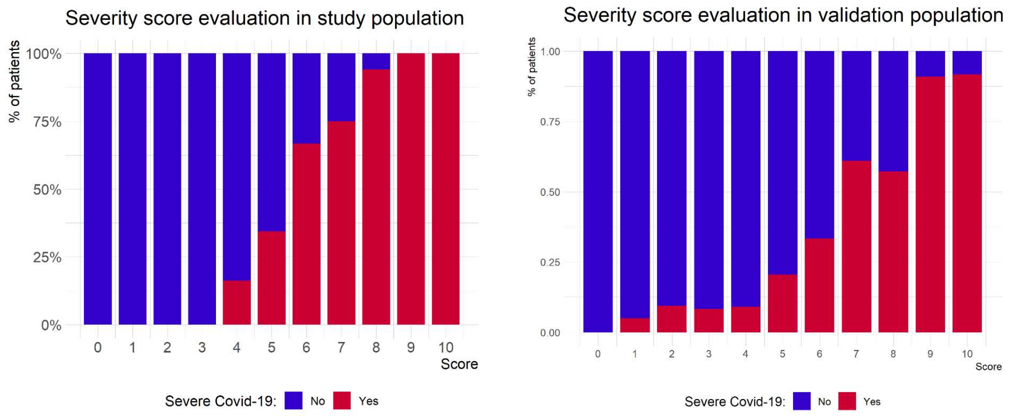

Figure S2: Percentage amount of patients with severe and non-severe COVID-19 in relation to score points in the study and validation cohort
