# Supplementary material for: Development and validation of a prognostic COVID-19 severity assessment (COSA) score and machine learning models for patient triage at a tertiary hospital
Source: J Transl Med. 2021 Feb 5;19:56. doi: 10.1186/s12967-021-02720-w (PMC7862984; doi:10.1186/s12967-021-02720-w)
Supplement: Supplementary file 3 — Additional file 3. External validation metrics. [file 12967_2021_2720_MOESM3_ESM.pdf]

Additional file 3

External validation metrics

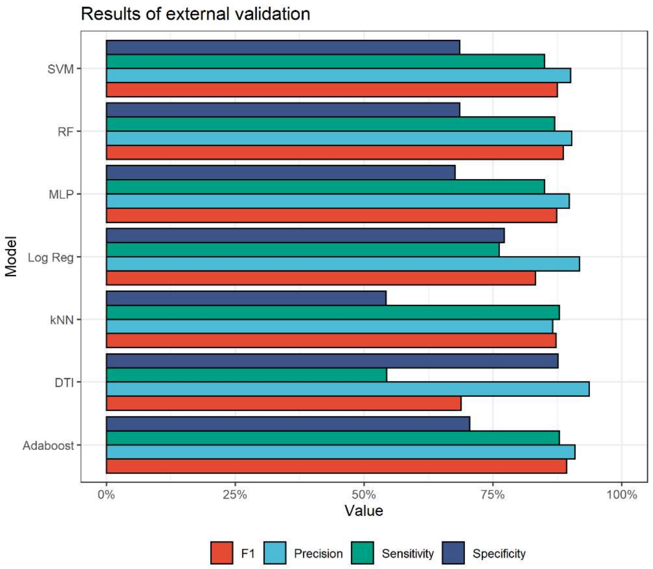

Figure S3: Comparison of the validation metrics of the external validation of the different machine learning models. *Sensitivity* measures the proportion of correctly identified true positives, *specificity* measures the proportion of true negatives, *precision* is the ratio of correctly predicted positive observations to the total predicted positive observations, and *F1* is the weighted average of precision and sensitivity.
